# Relationship between expression of cyclooxygenase 2 and neovascularization in human pterygia

**DOI:** 10.18632/oncotarget.22351

**Published:** 2017-11-09

**Authors:** Dongwei Liu, Chang Peng, Zhengxuan Jiang, Liming Tao

**Affiliations:** ^1^ Department of Ophthalmology, The Second Affiliated Hospital of Anhui Medical University, Hefei, Anhui 230601, PR China

**Keywords:** cyclooxygenase 2, angiogenesis, pterygium, immunohistochemical, VEGF

## Abstract

In present study, we are to test the relationship between cyclooxygenase 2 (COX-2) and angiogenesis in pterygium tissues from a group of Chinese patients. Here forty-five primary pterygium tissues and twenty-three normal bulbar conjunctival tissues were obtained during ophthalmologic surgeries. The primary pterygium samples were treated for the immunohistochemical evaluation of COX-2, CD31 and vascular endothelial growth factor (VEGF) antibodies for different tissues. In order to evaluate the relationship between COX-2 and neovascularization, a statistical analysis was performed using the SPSS 13.0 statistical software package. As results, in our study, 36 (80%) of the primary pterygia samples were found to be positive for COX-2 staining, which was not found in the normal conjunctivas. The density of the microvessels (MVD) was significantly increased in the COX-2 positive patients when compared to the COX-2 negative ones (19.06 ± 1.84 vs.10.44 ± 2.98, P=1.36×10^−5^) in the pterygia cases. In the group that was positive for COX-2, there were 39 (86.7%) samples with VEGF expression. Furthermore, the staining of both COX-2 and VEGF was localized to the lower and middle layers of the epithelium and the endothelial cells of the microvessels. When analyzed the relation between them, the expression of COX-2 showed a significant correlation with the MVD (P = 4.02×10^−4^) and VEGF (p = 2.72×10^−4^). In conclusion, the present study showed that COX-2 may play an important role in stimulating the angiogenesis of pterygium in concert with VEGF.

## INTRODUCTION

Human pterygium is considered to be one of the most common vision-threatening diseases among conjunctival disorders in ophthalmology. The clinical feature of this disease is a triangular or wing-shaped abnormal conjunctiva growing onto the cornea, which may induce irregular corneal astigmatism, even covering the entire pupil. Many studies reveal that certain factors are related to pterygium, including degeneration, hyperplastic properties, inflammatory features, and a luxuriant vasculature [1, 2]. Although the pathogenesis of pterygium has not been fully understood, several mechanisms may be able to explain the development of this disease, including the inflammatory process [3], anti-apoptotic mechanisms [4], cytokines [5], and angiogenic growth factors [6].

In previous studies, angiogenesis was thought to be the key initial event in the pathogenesis of pterygium [7], which is supported by the overexpression of the vascular endothelial growth factor (VEGF) found in pterygium. Additionally, many studies consider pterygium to be an ultraviolet radiation (UV)-related disease [8]. It is well known that cyclooxygenase 2 (COX-2) may be play the most important role in UV-related cutaneous carcinogenesis. Furthermore, the expression of cyclooxygenase is increased in pterygia [9, 10]. Additionally, several studies have reported that COX-2 is a key enzyme for cytokine-induced angiogenesis, and that it can regulate some angiogenic factors, such as VEGF in gastric and colon tumors [11, 12]. As UV is one of the most important factors in the pathogenesis of the pterygium, the COX-2 effect the functional roles in UV-related disease, and COX-2 has the role in regulation of VEGF, the relationship between the expression of COX-2 and neovascularization in human pterygia needs to clarify.

There have been few pathological studies concerning COX-2 mediated angiogenesis in pterygium tissues [9, 10]. Based on the expression of COX-2 elevated in pterygia, the function of COX-2 in UV-related disease, the aim of the present study was to investigate the expression of COX-2 in Chinese pterygium tissues, and evaluate a possible relationship between COX-2 and angiogenesis.

## RESULTS

### Immunostaining for COX-2, VEGF, and CD31 expression

**Figure 1:**
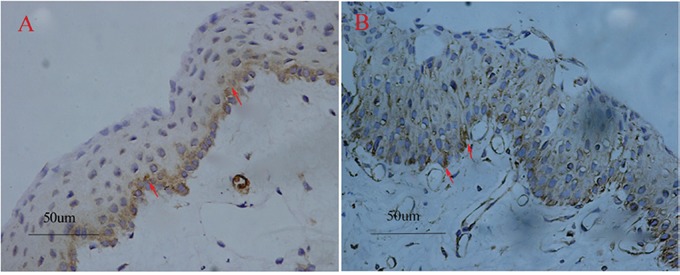
Pterygium tissues stained with COX-2 and VEGF The COX-2 positive staining was localized to the basal and middle layers of the epithelium and theinterstitium (red arrows) **(A)**. VEGF positive immunostaining case (red arrows) **(B)**. Original magnifications: A-B, 200×.

For this study, COX-2 positive staining was detected in 36 (36/45) primary pterygia. On the one hand, the expression of COX-2 was localized in the basal and middle layers of the epithelium in the cytoplasm of the cells, and in the endothelial cells of the vessels (Figure 1A). But on the other hand, the staining of the COX-2 protein was not observed in the normal conjunctiva.

39 of 45 (86.7%) samples stained positive for VEGF in the epithelium, stroma and vascular endothelial cells of the pterygium (Figure 1B). As a marker of the microvessels, CD31 was detected in all subjects. The mean value of the MVD in the pterygium was 18.13 ± 4.42, compared to 9.37 ± 2.81 per high power field in the normal conjunctiva with signal immunostaining.

### Relationship between COX-2 and microvessel density

**Figure 2:**
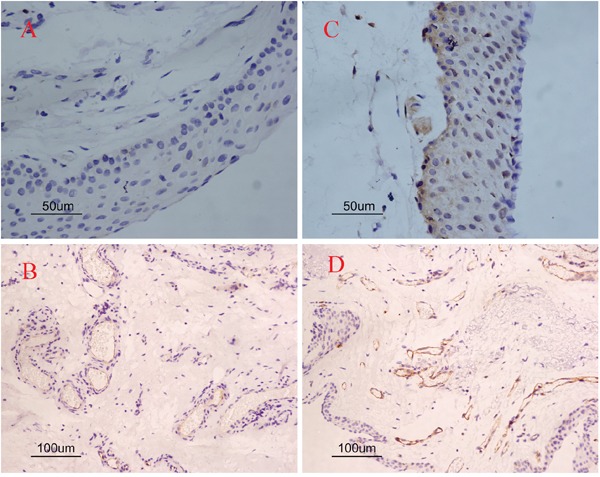
Single immunoreactivity stained with COX-2 and CD31 in pterygia Negative immunostaining cases **(A, C)**; positive immunostaining cases **(B, D)**. COX-2 negative immunostaining case (A) and COX-2 positive immunostaining case (C). Less positive staining of CD31 in COX-2 negative case (B) as compared to COX-2 positive immunostaining case (D). Original magnifications: A, C 200×; B, D 100×.

**Figure 3:**
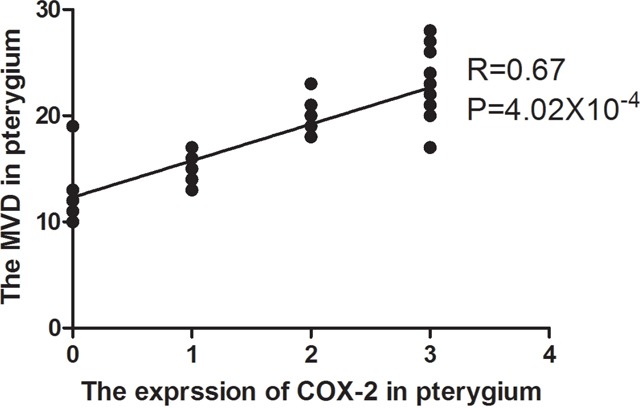
The linear regression map with the correlation between the expression of COX-2 and MVD The expression of COX-2 was scored as 0, 1, 2 and 3. MVD: microvessel density.

The expression of COX-2 and CD31 is summarized in Table 1. In pterygia, the mean MVD value of the COX-2 positive tissues was significantly higher than that of the COX-2 negative tissues (19.06 ± 1.84 vs. 10.44 ± 2.98, P=1.36×10^−5^; Figure 2A, 2C and Figure 2B, 2D). The logistic regression analysis showed that there was a significant correlation between the MVD and COX-2 in the tissues from the pterygia cases (r = 0.67, P = 4.02×10^−4^). The linear regression map with the correlation between the expression of COX-2 and MVD was showin Figure 3.

**Table 1:** Correlation between COX-2 and microvessel density in pterygium

Group	n	Microvessel Density ( Means±SD)	P
COX-2			
Negative	9	10.44±2.98	P=0.000
Positive	36	19.06±1.84	

Shown is the relationship between COX-2 and Microvessel Density. Microvessel Density expression was significantly increased in the group of pterygia with COX-2- Positive than COX-2- Negative immunostaining. Microvessel Density expression was significantly associated with COX-2 positivity. (P=0.000 assessed by Fisher's exact test).

### Relationship between COX-2 and VEGF

The relationship between COX-2 and VEGF is shown in Table 2. In the group of COX-2 positive pterygia tissues, 34 of 36 samples showed VEGF expression. The VEGF was significantly more frequently expressed in the COX-2 positive cases than in the COX-2 negative cases. The localization of COX-2 and VEGF was most frequently found at the basal and middle layers of the epithelium and in the endothelial cells of the microvessels (Figure 1A, 1B). The logistic regression showed that the expression of VEGF was correlated with COX-2 (r = 0.46, P = 2.72×10^−4^).

**Table 2:** Relationship between COX-2 expression and VEGF expression In pterygium

Group		COX-2		Total
		Negative	Positive	
VEGF	Negative	4	2	6
	Positive	5	34	39
Total		9	36	45

Shown is the relationship between COX-2 and VEGF. In the group of pterygia with cox-2 immunostaining, there were 34 (34/36, 94.4%) samples with VEGF expression. VEGF expression was significantly associated with COX-2 positivity (p<0.005 assessed by Fisher's exact test).

## DISCUSSION

Pterygium is one of the most common surface ocular lesions [1]. UV light was thought to be a triggering factor in the pathogenesis of pterygium, until now, but the mechanism of UV radiation in this disease is still unknown. Several studies have shown that UV-B irradiation can induce the expression of COX-2 through the activation of the NF-KB signaling pathway in tumor formation and growth [14, 15]. Therefore, COX-2 has been regarded as a crucial factor in UV-related carcinogenesis. Several studies showed that the expression of COX-2 and VEGF were found to be increased in pterygia, when compared to normal conjunctival and subconjunctival tissue [16, 17]. Based on these information, it is reasonable to hypothesize the following mechanism in the pathogenesis of pterygium that UV-B irradiation causes the activation of the NF-KB signaling pathway and the expression of COX-2 and VEGF elevating, the higher levels of COX-2 and VEGF could cause the angiogenesis, and then lead to pterygium.

In our study, COX-2 was expressed in 80% of the pterygium specimens, and no immunoreactivity samples were detected in the normal conjunctival and subconjunctival samples. The percentage of pterygia samples staining positively for COX-2 in this study is similar to the percentages described elsewhere [9, 10]. The expression of COX-2 was localized in the basal and middle layers of the epithelium in the cytoplasm of the cells and in the endothelial cells of the vessels. This result was consistent with a previous study indicating the localization of the COX-2 positively staining cells in the basal and middle layers of the pterygia epithelium [10]. These results support the pivotal relationship between COX-2 and pterygium, and provided molecular evidence for the effects of UV radiation in this disease.

Angiogenesis is an important pathological characteristic in the development of pterygium. Furthermore, many studies have reported that COX-2 could enhance angiogenesis in several types of tumors. These results have also been reported in gallbladder and gastric adenocarcinomas. *In vivo* researches have suggested that anti-COX-2 treatment may result in the reduction of angiogenesis expression [18]. As a UV-related tumor, there are few research studies examining this relationship between COX-2 and neovascularization in human pterygium tissue. This is the first study examining the relationships between COX-2, VEGF, and the microvessel density in pterygia.

In this study, we found that the MVD was higher in the COX-2 positive cases when compared to the COX-2 negative cases, and a correlation indeed existed between COX-2 and MVD in pterygia. VEGF, TNF-α, PDGF, and b-FGF are involved in the pathogenesis of neovascularization [19], and VEGF, which could be up-regulated by UVB, seems to be one of the most important regulators of the angiogenic mediators [20, 21]. Many studies have revealed that VEGF caused capillary tube formation in co-cultures [22]. Furthermore, *in vitro* research has confirmed that COX-2 overexpression is associated with increased levels of RNA for angiogenic factors, including VEGF in cultured human umbilical vein endothelial cells (HUVECs) [19]. Therefore, in order to further illustrate the point of COX-2 promoting angiogenesis in pterygium tissues, we also tested the correlation between COX-2 and VEGF by immunohistochemical means. The localization of these two proteins was frequently found at the basal and middle layers of the epithelium and the endothelial cells of the microvessels. A significant correlation between COX-2 upregulation and VEGF expression was proven in our study, and this result was in agreement with previous data about the positive correlation of the two proteins in the diseases of human gastric adenocarcinoma and colon cancer cell lines [23, 24]. However, the results were not agreement with a previous study reporting that VEGF expressing vascular endothelial cells were mostly negative for COX-2 expression [25].

VEGF is not only found in resident cells (epithelial cells, fibroblasts, and the endothelial cells of microvessels) that play an important role in COX-2 expression, but also in invading inflammatory cells like macrophages [25], and it is important to identify the cell types staining positively for COX-2 and VEGF. The number of macrophages is very low in pterygia, and the COX-2 and VEGF are highly expressed in the endothelial cells of the microvessels. Although this study has not identified the cell types that could secrete COX-2 and VEGF, we can speculate that the COX-2 and VEGF were secreted by the endothelial cells of the microvessels. The different results of the two studies may be caused by the different status and different ethnicity of the patients. Based on these, we could suggest that COX-2 may promote neovascularization in the pathogenesis of pterygia, to a certain degree, in Chinese patients.

This study does have some limitations. Firstly, the specific mechanism of COX-2 promoting the neovessels was not fully understood. Secondly, all of the subjects came from the Chinese Han population, and these results should be tested in other populations.

In conclusion, this study is the first to report a significant correlation between COX-2 upregulation and neovascularization in pterygium. The existing relationship confirmed that COX-2 plays an important role in the process of induced angiogenesis. Furthermore, COX-2 inhibitors may be regarded as one of the most valuable treatments for pterygium.

## MATERIALS AND METHODS

### Patients and study design

The study group for this research included 45 primary pterygium samples and 23 normal conjunctival and subconjunctival samples which were recruited from patients at the Second Hospital Affiliated of Anhui Medical University. All pterygium patients (21 males and 24 females) underwent excision via the bare scleral technique. The average age of the patients was 56.2 ± 11.2 years, and the age ranged from 31 to 73. Normal conjunctive samples were obtained from individuals (12 males and 11 females) with an age range from 37 to 78 and an average age of 66.9 ± 9.5 years, who underwent strabismus surgery without pterygia. All patients underwent a complete ophthalmic examination. With the exception of a topical anesthetic, none of the patients received any medication for the surgery, and no drugs or chemical agents were used during the surgical operation. This study was carried out with the approval of the Ethics Committee of Anhui Medical University. Complete information on the patients was available for evaluation.

### Immunohistochemistry

All specimens were fixed in cold 10% formalin, and embedded in paraffin. Next, 3 μm sections were cut, mounted on glass and dried overnight at 37°C for immunofluorescence analysis. The tissue sections were sequentially deparaffinized in xylene, rehydrated in alcohol, washed in phosphate-buffered saline and heated twice in a microwave oven for 5 min in citrate buffer (pH 6.0) for antigen retrieval, in order to enhance the immunoreactivity of the target antibody. To block nonspecific binding of the secondary antibody, the sections were incubated with goat serum for 30 minutes, and then immerged with the primary antibodies at 4°C overnight. Anti-VEGF (1:150; Santa Cruz Biotechnology Inc., Santa Cruz, CA), rabbit polyclonal anti-CD31 antibody (ready to use; Zhongshan Biologic and Technical Company, Beijing, China), and polyclonal anti-COX-2 antibody (RS-0732 R; Zhongshan Biologic and Technical Company, Beijing, China) were used as the primary antibodies. The procedure was then followed by a conventional streptavidin peroxidase method, based on the manufacturers’ instructions, where the signals were developed with 3, 3′-diaminobenzine (DAB) for 5 min and counter-stained with hematoxylin.

### Evaluation of immunoreactivity

The immunoreactive cells of VEGF were simultaneously assessed by two investigators, without knowledge of the clinical data, to evaluate the staining under a light microscope (80i, Nikon, Tokyo, Japan). The results of the expression of VEGF were based on a semi-quantitative score for the percentile quadrants of the positive cells: 0%, no expression; 1% - 25%, score +; 26% - 50%, score++; > 50% score +++. Scores of +, ++, and +++ were considered to be as positive immunostaining or described elsewhere, and the score of 0 was considered to be negative immunostaining.

The results were scored for the percentage of positive staining for COX-2 as: score 0, no positive staining; score +, from 1% - 10%; score ++, from 11% - 50%; score +++, more than 50% positive cells. In this study, scores of +, ++, and +++ were considered to be positive immunostaining, and a score of 0 was seen as negative immunostaining.

The patients were divided into four groups: COX-2 positive, COX-2 negative, VEGF positive, and VEGF negative. Microvessel counts were achieved by using the method described by Domenico et al. [13]. Two investigators assessed the samples in the condition without knowledge of the final pathological diagnosis, and five fields (200×) per sample were examined with a light microscope (80i, Nikon, Tokyo, Japan). Either without or with a thin basement membrane, the CD31 positive capillaries and small venules were identified as transversally sectioned tubes, with a single layer of endothelial cells. Each assessment was agreed upon in turn. Microvessels were counted with a planimetric point count method, accordingly this method, only the microvessels transversally cut and occupying the reticulum points were counted. Since almost the entire section was analyzed for each sample, and transversally sectioned microvessels hit the intersection points randomly, the method allowed for objective counts. The mean ± SD (standard deviation) and medians were determined for each section, sample, and group of samples.

### Statistical analysis

The data analysis was conducted using SPSS 13.0 (Statistical Package for Social Sciences; SPSS Inc., Chicago, IL), and a p-value of less than 0.05 was accepted as statistically significant. The Wilcoxon rank sum test and χ^2^ test or Fisher's exact test were used to detect the data. When the qualification of the χ^2^ test was not satisfied, a continuity correlation was used to modify the results. Logistic regression analysis was used to estimate the relation between the expression of COX-2 and MVD, VEGF (version 10.0; SPSS Inc.).
